# The association between early neurological deterioration and whole blood purine concentration during acute stroke

**DOI:** 10.1186/s40364-019-0158-y

**Published:** 2019-04-03

**Authors:** Alexander J. Martin, Nicholas Dale, Christopher H. E. Imray, Christine Roffe, Craig J. Smith, Faming Tian, Christopher I. Price

**Affiliations:** 1 NIHR Newcastle Biomedical Research Centre and Institute of Neuroscience, Newcastle University, Newcastle upon Tyne, UK; 2 School of Life Sciences, University of Warwick, Coventry, UK; 3 Coventry and Warwickshire County Vascular Unit, University Hospitals Coventry and Warwickshire NHS Foundation Trust, Coventry, UK; 4 Institute for Science and Technology in Medicine, University of Keele, Stoke-on-Trent, UK; 5 Division of Cardiovascular Sciences, University of Manchester, Manchester, UK; 6 Sarissa Biomedical Ltd, Coventry, UK; 7 Stroke Research Group, Newcastle University, Newcastle Upon Tyne, NE2 4AE UK

**Keywords:** Stroke, Cerebrovascular disease, Deterioration, Purines, Penumbra

## Abstract

**Background:**

Early neurological deterioration (END) is common after stroke. Prediction could identify patients requiring additional monitoring and intervention. Purines, breakdown products of adenosine triphosphate which accumulate during acute hypoxia, may reflect the subclinical presence of vulnerable tissue. We considered whether whole blood purine concentration (WBPC) measurements during acute stroke were associated with subsequent END.

**Methods:**

Patients within 4.5 h of stroke onset underwent point-of-care finger-prick measurement of WBPC and blinded assessment of symptom severity using the National Institutes of Health Stroke Scale (NIHSS). END was defined as an NIHSS increase ≥2 points at 24–36 h compared to baseline.

**Results:**

15/152 (9.8%) patients experienced END with a median [IQR] NIHSS increase of 4 [2–7] points. There were no strong associations between END and baseline NIHSS, clinical stroke subtype, thrombolytic therapy, physiological characteristics or time to assay. The median [IQR] WBPC concentration (uM) was higher before the occurrence of END but without statistical significance (7.21 [4.77–10.65] versus 4.83 [3.00–9.02]; *p* = 0.1). Above a WBPC threshold of 6.05uM, the risk of END was significantly greater (odds ratio 3.7 (95% CI 1.1–12.4); *p* = 0.03).

**Conclusion:**

Although the study lacked statistical power, early WBPC measurement could be a convenient biomarker for identifying acute stroke patients at risk of END, but further evaluation is required.

## Background

Early neurological deterioration (END) describes worsening of symptoms during the initial period following stroke onset and is associated with poorer outcomes. Incidence ranges from 2.2 to 37.5% depending upon the choice of severity threshold and the time elapsed between assessments [1–4]. A typical definition is an increase of at least two points in National Institutes of Health Stroke Score (NIHSS) at 24–48 h following admission [1, 2]. Secondary causes are common (e.g. sepsis), but END also reflects progressive failure of penumbral tissue surrounding an ischaemic core or compressive haematoma [3–5], as well as local haemodynamic disturbances, extension of thrombosis, excitotoxicity, and inflammation, which may be greater influences upon the progression of lacunar infarction [6]. Although advanced imaging techniques such as MR and CT perfusion can demonstrate the volume of threatened ischaemic tissue during acute stroke, this is not quantifiable using standard brain imaging and advanced techniques are not routinely available [5]. Convenient prediction of END risk during initial clinical review could aid identification of patients requiring additional imaging, monitoring and/or early intervention.

Purines are nitrogen-containing compounds with a short half-life which are released by breakdown of adenosine triphosphate (ATP) and consumed again during oxidative phosphorylation. Using a novel biosensor [7] to quantify purines (adenosine, inosine and hypoxanthine), it has been demonstrated that carotid clamping during endarterectomy without general anaesthetic results in rapid elevation of the whole blood purine concentration (WBPC) from a baseline mean of 2.4 μM (95% CI 1.3–4.0) to 6.7 μM (4.7–11.5), quickly returning to 1.9 μM (1.3–4.0) following clamp removal, without neurological sequelae [8]. In the context of acute stroke, WBPC may therefore be a responsive biomarker which rises not only in proportion to the extent of clinically symptomatic hypo-perfused tissue, but also according to the volume of subclinical penumbra prior to its eventual re-oxygenation or infarction. Development of the bio-sensor into a rapid point-of-care test provided the opportunity to perform a double-blind observational cohort study exploring whether early WBPC measurement was associated with subsequent END amongst patients with very recent stroke symptoms.

## Methods

Adults within 4.5 h of stroke symptom onset at 4 hospitals underwent point-of-care capillary sampling of WBPC (SMARTChip assay; Sarissa Biomedical Ltd., Coventry, UK), clinical assessment (physiological observations; NIHSS) and CT brain imaging [9]. There was no restriction on the type of symptoms, but NIHSS was always > 0 at the time of sampling. Patients receiving chemotherapy and acute gout treatment were excluded.

Stroke diagnosis and clinical subgroup using the Oxford Community Stroke Project classification [10] were confirmed by a local specialist following clinically appropriate investigations, with additional adjudication by at least one study investigator. Patients with subarachnoid haemorrhage were excluded. The WBPC reading was concealed from clinical assessors and performed before any emergency medical treatment. All patients receiving intravenous thrombolysis underwent repeat brain imaging at 24 h. END was defined as an increase ≥2 points between the first NIHSS and a second assessment 24–36 h later.

Statistical comparison was made using Fisher’s Exact Test for categorical data and the Mann Whitney U Test for continuous variables due to the small sample size and data distribution. Independent samples T-Test was also applied to the WBPC values. Spearman’s rho examined correlation between variables. The utility of WBPC as a biomarker for END was assessed by the area under the curve (AUC) of the resulting receiver operating characteristic (ROC) plot. Ethics approval was granted by the National Research Ethics Service in England (reference 16/WM/0164). All participants or their personal consultee gave informed consent consistent with the principles of the Declaration of Helsinki.

## Results

One hundred fifty-two patients were enrolled: 66 ischaemic stroke who did not receive intravenous thrombolysis (ISNT), 61 ischaemic stroke receiving intravenous thrombolysis (IST) and 25 intracerebral haemorrhage (ICH). No patients received intra-arterial treatment. For 9 of 66 ISNT cases, the NIHSS at 24 h was zero and the discharge diagnosis was transient ischaemic attack (TIA). Early neurological deterioration occurred in 15 (9.8%) cases: 4 ISNT, 5 IST (none experienced haemorrhage on repeat brain imaging) and 6 ICH (1 requiring craniotomy). Their median NIHSS increase was 4 (IQR 2–7) points. Apart from a small difference in oxygen saturations favouring the END group, characteristics were similar between groups (Table 1).

**Table 1:** Characteristics and outcomes with and without early neurological deterioration

	non-END (*n* = 137)	END (*n* = 15)	*p*-value
Female, n (%)	70 (51%)	9 (60%)	0.59
Age (years)	76.0 (65.0–82.0)	79.0 (68.5–86.0)	0.42
Systolic blood pressure (mmHg)	158 (138–191)	174 (138–183)	0.82
Diastolic blood pressure (mmHg)	87 (75–96)	81 (74–92)	0.32
Heart rate (per minute)	83 (72–92)	76 (60–91)	0.27
Respirations (per minute)	17 (16–18)	16 (15–18)	0.89
Temperature (°C)	36.4 (36.1–36.7)	36.5 (36.4–36.8)	0.43
Oxygen saturations (%)	96 (95–98)	97 (96–99)	0.06
Clinical subgroup			
LAC ischaemia/haemorrhage (subtotal %)	31/6 (27%)	2/2 (27%)	0.98
PAC ischaemia/haemorrhage (subtotal %)	39/5 (32%)	4/0 (27%)	0.67
TAC ischaemia/haemorrhage (subtotal %)	31/6 (27%)	3/4 (46%)	0.11
POC ischaemia/haemorrhage (subtotal %)	8/2 (7%)	0/0 (0%)	0.28
Transient ischaemic attack^a^	9 (7%)	–	–
Intravenous thrombolysis treatment (%)	56 (41%)	5 (33%)	0.59
Baseline NIHSS	7 (4–14)	13 (6–17)	0.26
24-36 h NIHSS	4 (1–10.25)	18 (13–25)	< 0.01
Minutes from onset to assay	133 (96–180)	104 (73–146)	0.15
Whole blood purine concentration (μM)	4.83 (3.00–9.02)	7.21 (4.77–10.65)	0.10
Mean [SD] whole blood purine concentration (μM)	7.65 [7.67]	11.3 [11.60]	0.26

^a^Transient ischaemic attack cannot co-exist with END. Variables are median (IQR) unless otherwise stated; *END* early neurological deterioration, *NIHSS* National Institutes of Health Stroke Scale, *LAC* lacunar circulation, *PAC* partial anterior circulation, *TAC* total anterior circulation, *POC* posterior circulation

Baseline NIHSS was higher amongst the END group, but this was not statistically significant (*p* = 0.26), and no correlation was found between baseline NIHSS and WBPC overall (Spearman’s rho = − 0.126; *p* = 0.313). No significant differences were observed in the distribution of clinical subgroups, including lacunar stroke.

Overall, WBPC was higher for the END group, but this did not reach statistical significance. However, above an optimal ROC threshold of 6.05 μM for WBPC, the risk of END was significantly greater (odds ratio 3.7 (95% CI 1.1–12.4); *p* = 0.03). The ROC AUC for prediction of END using a WBPC threshold of 6.05 μM was 0.63 (95% CI: 0.49–0.77).

**Fig. 1:**
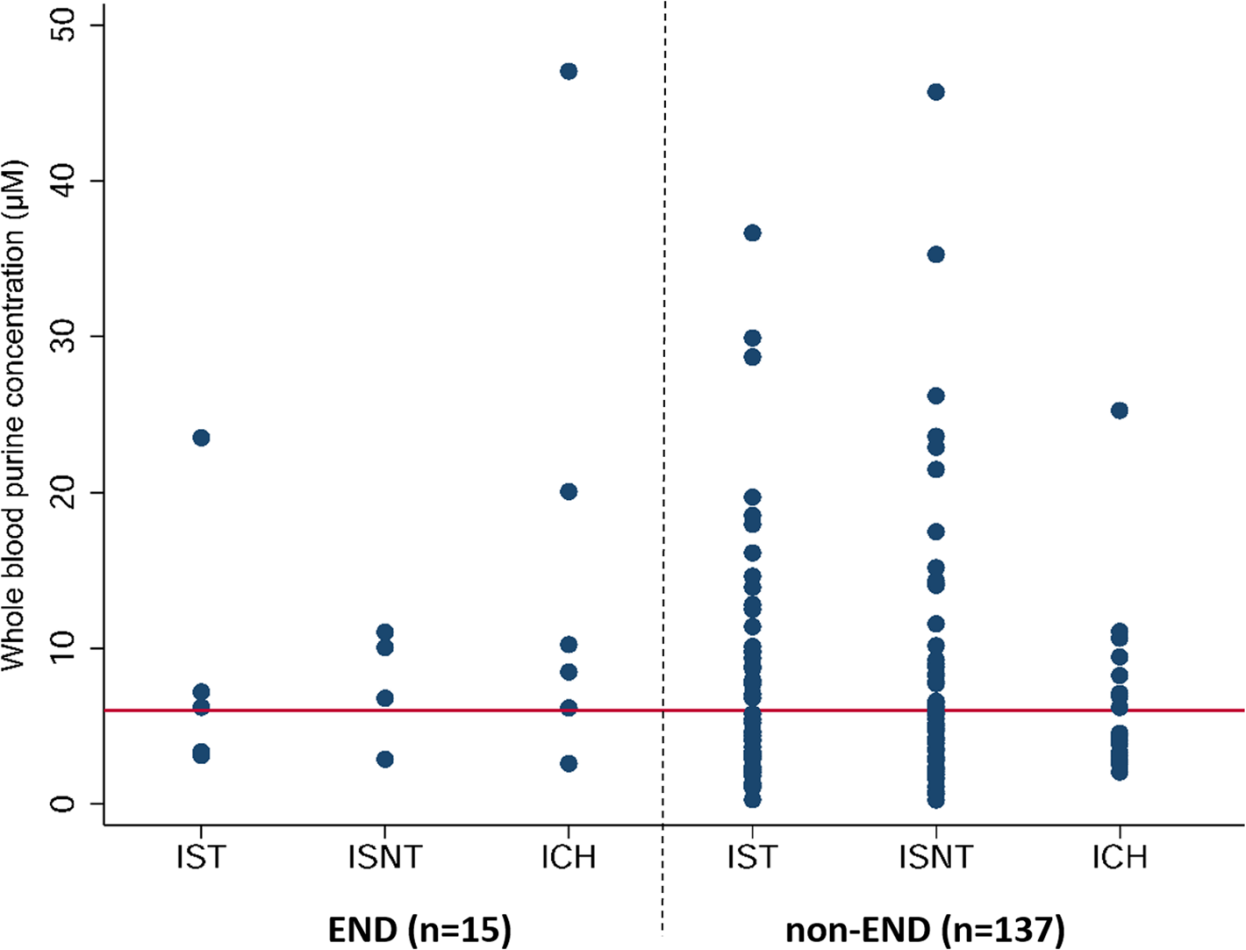
Whole blood purine concentration according to clinical diagnostic group. IST: ischaemic stroke (thrombolysis); ISNT: ischaemic stroke (no thrombolysis); ICH: intracerebral haemorrhage; END: early neurological deterioration. The horizontal line shows an optimised diagnostic threshold of 6.05μM

Figure 1 shows the distribution of individual WBPC measures according to each clinical diagnostic group +/− END at a threshold of 6.05 μM. There were no statistically significant WBPC differences between IST, ISNT and ICH groups.

## Discussion

Elevated WBPC during the first 4.5 h after stroke onset was weakly associated with a greater risk of END at 24–36 h. Although the association did not reach statistical significance due to the size of the END group and the mixed cohort characteristics, this remains an interesting preliminary finding. As measurements occurred before the typical timing of secondary causes such as sepsis, and there were no major physiological differences between groups on admission, early WBPC elevation may indicate the subclinical presence of vulnerable tissue. However, the moderate size of the AUC reflects the preliminary nature of this study, and it would be necessary to undertake an adequately powered diagnostic accuracy trial, which actively seeks multiple causes for END over a longer interval, to be confident that WBPC is a biomarker capable of informing early clinical decisions about the intensity of monitoring and thresholds for additional treatment.

A maximum symptom duration of 4.5 h was chosen to reflect the time window used by most clinical services to trigger a standard emergency response for suspected stroke, thereby ensuring that study results were directly relevant to the largest and most important patient population. In addition, extension of the sampling time beyond 4.5 h would have demanded a larger overall number of participants to detect any relationship between WBPC and END as the probability of viable penumbral tissue would be reduced, whilst risks would increase for the occurrence of other conditions potentially affecting WBPC results (e.g. sepsis from early aspiration pneumonia).

It is important to acknowledge that the END group displayed greater initial stroke severity, which could partly explain their increased risk of deterioration (e.g. due to cerebral oedema). However, NIHSS comparison with the nEND group was not statistically significant, and no relationship was found between baseline NIHSS and WBPC values overall. The latter observation may reflect a cellular mechanism whereby tissue which has become irreversibly damaged will cease ATP metabolism and hence purine production, whereas hypoxic areas at risk will consume ATP but without replenishment, thereby elevating WBPC until perfusion is restored or cell death occurs. A similar observation was previously described during temporary carotid clamping, where the WBPC rose above the current END threshold to a mean of 6.7 μM, but returned to baseline levels without any neurological consequences when perfusion was restored [8]. A future study of serial measurements amongst patients with different baseline NIHSS +/− thrombolysis (e.g. every 6 h for 48 h) would assist with understanding the dynamic profile of WBPC in relation to the evolution of stroke, and provide greater confidence that there is a single threshold which reliably indicates ongoing penumbral ischaemia rather than irreversible tissue injury.

As this study was an initial exploration of the relationship between WBPC and END, it was not designed to specifically consider mechanisms for deterioration in different clinical subgroups e.g. small versus large vessel stroke, although none were evident. Failure of ATP production is likely to be a common end-point for all mechanisms leading to END, and no clear association might be expected with WBPC elevation other than to reflect the rate at which tissue injury occurs. However, as previously described, the frequency of END was higher amongst the ICH than the IS group (6/25 versus 9/127 cases) [1, 4, 11]. Since clinical trial evidence has confirmed a relationship between high blood pressure, haematoma expansion and neurological outcome, future studies exploring the role of END biomarkers should also consider haematoma volume and physiological data collected during the first 24 h after onset [4, 11, 12].

For patients with ischaemic stroke, the value of biomarkers as indicators of subclinical penumbral volume would be best demonstrated by simultaneous MR or CT perfusion imaging, which are increasingly used to confirm the presence of tissue that might still be recovered by mechanical thrombectomy [13]. If a strong correlation was found between core:penumbra mis-match and WBPC, this could indicate a potential role as a simple screening test to identify patients during the acute phase of ischaemic stroke who should undergo advanced imaging, particularly when the symptom onset time is unclear.

An additional limitation of our study was collection of NIHSS data from each patient by a single assessor, but all had completed standard training and were unaware of the WBPC result. A sensitive definition for END was applied and although there may be little clinical benefit derived from identifying patients at risk of mild deterioration, the observed median NIHSS increase of 4 points mirrors the most commonly employed definition for symptomatic ICH following thrombolysis.

## Conclusions

Early WBPC measurement could be a useful point-of-care biomarker for identifying patients at risk of END. However, due to the small size of this mixed cohort and the sensitive definition used, further research is required with a larger population which incorporates perfusion imaging and physiological data to determine how WBPC relates to stroke pathophysiology, and the added value of measurement for clinical decision making.

## Abbreviations

ATP: Adenosine triphosphate
AUC: Area under the curve
END: Early neurological deterioration
ICH: Intracerebral haemorrhage
IS: Ischaemic stroke
ISNT: Ischaemic stroke non-thrombolysis
IST: Ischaemic stroke thrombolysis
nEND: Non-early neurological deterioration
NIHSS: National Institutes of Health Stroke Scale
ROC: Receiver operating characteristic
TIA: Transient ischaemic attack
WBPC: Whole blood purine concentration
